# *Euphorbia fanjingshanensis* (Euphorbiaceae), a new species from section *Helioscopia* in southwestern and central China

**DOI:** 10.3897/phytokeys.277.187415

**Published:** 2026-07-16

**Authors:** Ya-Lin Feng, Lei Shi, Ranto Tiana Ratsiferanarivo, Xiang-Dong Qiu, Zhi-Hao Xin, Hai-Yang Yao, Sheng-Wei Wang, Neng Wei, Qing-Feng Wang

**Affiliations:** 1 State Key Laboratory of Plant Diversity and Specialty Crops, Wuhan Botanical Garden, Chinese Academy of Sciences, Wuhan 430074, Hubei, China Guizhou Fanjingshan Forest Ecosystem Observation and Research Station, Fanjingshan National Nature Reserve Administration Tongren China; 2 University of Chinese Academy of Sciences, Beijing 100049, China State Key Laboratory of Plant Diversity and Specialty Crops, Wuhan Botanical Garden, Chinese Academy of Sciences Wuhan China; 3 Guizhou Fanjingshan Forest Ecosystem Observation and Research Station, Fanjingshan National Nature Reserve Administration, Tongren 554400, Guizhou, China Department of Plant Biology and Ecology, Faculty of Sciences, University of Antananarivo Antananarivo Madagascar; 4 Department of Plant Biology and Ecology, Faculty of Sciences, University of Antananarivo, Antananarivo 101, Madagascar School of Ecology and Environment, Tibet University Lhasa China; 5 School of Ecology and Environment, Tibet University, Lhasa 850032, Xizang, China University of Chinese Academy of Sciences Beijing China; 6 Sino-Africa Joint Research Center, Chinese Academy of Sciences, Wuhan 430074, Hubei, China Sino-Africa Joint Research Center, Chinese Academy of Sciences Wuhan China

**Keywords:** Leafy spurges, morphology, phylogeny, subgenus *Esula*, taxonomy

## Abstract

*Euphorbia fanjingshanensis*, a new species from southwestern and central China, is described and illustrated. Morphologically, it is most similar to *E. micractina* and *E. hylonoma* in E. subg. Esula sect. *Helioscopia*, but can be readily distinguished by its lanceolate leaves with acute apices, reddish to crimson peduncles, and tuberculate capsules, and ovoid-globose dark brown seeds (ca. 2.5 × 2 mm). Phylogenetic analyses based on combined nuclear ITS and plastid *ndhF* sequences further confirm its placement within the clade of *E.* sect. *Helioscopia*, and reveal a close relationship with other species of the East Asian Clade.

## Introduction

*Euphorbia* in Linnaeus (1753: 560) (Euphorbiaceae) is one of the largest and most morphologically diverse genera in angiosperms, comprising more than 2,000 species distributed nearly worldwide (Steinmann and Porter 2002; Horn et al. 2012; Webster 2014). Because of its extensive homoplasious characters, as well as the tremendous species diversity and wide distribution, the taxonomy of genus *Euphorbia* has long been a hard nut (Webster 1967; Steinmann and Porter 2002; Bruyns et al. 2006). Integrative studies combining morphology and molecular phylogeny have greatly improved species delimitation and classification within the genus (Yang et al. 2012; Dorsey et al. 2013; Peirson et al. 2013; Riina et al. 2013).

Within *Euphorbia*, subgenus *Esula* Pers. in Persoon (1806: 14) mainly includes temperate perennial herbs characterized by leafy stems, cyathia with glands lacking appendages, and tuberculate or verrucose fruits (Riina et al. 2013). Section *Helioscopia* Dumort. in Dumortier (1827: 87) shows extensive variation in vegetative and reproductive characters. Molecular evidence supports that East Asian members of this section, such as *E. micractina* Boiss. in Boissier (1862: 127), *E. hylonoma* Hand.-Mazz. in Handel-Mazzetti (1931: 230) and *E. pekinensis* Rupr. in Ruprecht (1859: 239), form a distinct clade corresponding to the Sino-Japanese element and Sino-Himalayan element in the East Asian Group (Geltman 2015; Yu et al. 2022).

China harbors about 46 species from Euphorbia subg. Esula, including several recently described taxa such as *Euphorbia xianxialingensis* (Zhang et al. 2020), *Euphorbia mongoliensis* (Zang et al. 2021), and *Euphorbia xiangxiui* (Yu et al. 2022). However, the diversity of this group remains poorly explored. During investigation trips to Fanjingshan National Nature Reserve, Guizhou Province, we discovered two distinct populations of *Euphorbia* growing in humid and open rocky slopes. The same species was later documented in Chuankongdong Cave (Yichang, Hubei Province), in Langao County (Shaanxi Province) as well. Largely due to tuberculate fruits and cyathial glands without appendages, this *Euphorbia* is readily placed in E. subg. Esula sect. *Helioscopia*, which was defined by Riina et al. (2013). It resembles *E. micractina* and *E. pekinensis* in general habit but differs in having lanceolate leaves with acute apices, reddish to crimson peduncles, moderately tuberculate capsules, and dark brown ovoid-globose seeds. Phylogenetic analyses based on combined nuclear ITS and plastid *ndhF* sequences support this *Euphorbia* as an independent lineage within *E.* sect. *Helioscopia*. Here we describe it as a new species, *E. fanjingshanensis*.

## Materials and methods

### Morphological analyses

Voucher specimens of the new species were collected and prepared during fieldwork. Morphological features were observed, measured using Adobe Photoshop 2020, and photographed with a Nikon D800E camera and a TS-10W zoom stereo microscope. The whole plant, synflorescence, leaves, and dissected flowers were carefully documented. The morphological description of *E. fanjingshanensis* was derived from the observations and measurements on living plants, and designated type specimens preserved at HIB. Comparisons of morphological characters with other related *Euphorbia* species were implemented based on the examination of digitized images from online sources such as Chinese Virtual Herbarium (https://www.cvh.ac.cn/index.php), the Global Plants on JSTOR (https://plants.jstor.org/) and Plant Photo Bank of China (https://ppbc.iplant.cn/), as well as the descriptions in floras and journals. Botanical terminology follows the standards of the Kew Plant Glossary (Beentje 2010).

### Phylogenetic sampling

To determine the phylogenetic placement of the new species within *Euphorbia*, we employed two molecular markers: the nuclear ribosomal Internal Transcribed Spacer (ITS) region, and the plastid *ndhF* gene. Both markers have been widely used in systematic and phylogenetic studies of *Euphorbia* (Bruyns et al. 2006; Zimmermann et al. 2010; Yang et al. 2012; Yu et al. 2022). A total of 82 samples representing 79 species were included for phylogenetic reconstruction, representing *Euphorbia* sect. *Helioscopia* and its closely related sections *E.* sect. *Lathyris*, *E.* sect. *Lagascae*, and *E.* sect. *Holophyllum*, which were selected as outgroups according to previous phylogenetic frameworks (Riina et al. 2013; Yu et al. 2022). Details of all sampled taxa and their corresponding GenBank accession numbers are provided in Table 1. In this study, two ITS and two *ndhF* sequences were newly generated, while 76 ITS and 36 *ndhF* sequences were obtained from the NCBI GenBank database. Genomic DNA extraction and amplification of the ITS and *ndhF* regions followed the protocols described by Barres et al. (2011) and Riina et al. (2013). Primer design, PCR amplification, and sequencing were performed as outlined in Riina et al. (2013).

**Table 1.** List of taxa sampled with information related to voucher specimens, GenBank accession numbers, and references (– indicates the missing information).

**Specie**s	**Voucher**	**ITS**	***ndhF***	**Reference**
*Euphorbia acanthothamnos*	*R. Riina 1563* (MICH)	JQ750879	JQ750756	Yang et al. 2012
*E. adenochlora*	*Oita 01393* (SAPS)	KC212161	–	Riina et al. 2013
*E. akenocarpa*	*L. Barres et al. BCN53041* (*duplicate*) (MICH)	JN250108	JN249098	Horn et al. 2012
*E. alatavica*	*G. Lazkov s.n*. (LE)	GU953741	–	Kryukov et al. 2010
*E. alpina*	*P. Berry 7980* (MICH)	KC212167	–	Riina et al. 2013
*E. alta*	*A. Sanders 5905* (RSA)	AF537553	–	Steinmann and Porter 2002
*E. altaica*	*D. Geltman 143* (LE)	KC212169	–	Riina et al. 2013
*E. angulata*	*J. Esser 06–26* (M)	KC212175	KC212441	Riina et al. 2013
*E. apios*	*W. Gutermann 25543* (Herb. Gutermann)	JN010027	–	Frajman and Schönswetter 2011
*E. arguta*	*R. Meikle 2237* (LE)	KC212176	–	Riina et al. 2013
*E. aristata*	*D. Geltman s.n*. (LE)	GU979434	–	Kryukov et al. 2010
*E. austriaca*	*B. Frajman & P. Schönswetter 11382* (IB)	JN010028	–	Frajman and Schönswetter 2011
*E. buchtormensis*	*S. Smirnov s.n*. (LE)	KC212196	–	Riina et al. 2013
*E. capitulata*	*M. Turjak & B. Frajman 11838* (IB)	JN010032	–	Frajman and Schönswetter 2011
*E. carniolica*	*M. Turjak & B. Frajman 11836* (IB)	JN010033	–	Frajman and Schönswetter 2011
*E. carpatica*	*A. Csergö 12463* (IB)	JN010034	–	Frajman and Schönswetter 2011
*E. cashmeriana*	*L. Edelberg 781* (W)	HQ900592	–	Barres et al. 2011
*E. ceratocarpa*	*R. Vilatersana et al. 1141* (*duplicate*) (BC)	KC212203	KC212468	Riina et al. 2013
*E. clementei*	*J. Molero BCN49327* (BCN)	HQ900596	–	Barres et al. 2011
*E. condylocarpa*	*Y. Salmaki et al. 39955* (TUH)	KC212208	KC212473	Riina et al. 2013
*E. coniosperma*	*L. Vakhtina s.n*. (LE)	KC212209	–	Riina et al. 2013
*E. corallioides*	*R. Vilatersana et al. 1161* (BC)	HQ900597	–	Barres et al. 2011
*E. depauperata*	*J. Morawetz 466* (MICH)	KC212225	–	Riina et al. 2013
*E. dimorphocaulon*	*R. Riina 1673* (duplicate) (MA)	JN250137	–	Horn et al. 2012
*E. dulcis*	*J. Molero & L. Saéz BCN54322* (BCN)	KC212230	KC212495	Riina et al. 2013
*E. dumalis*	*J. Aldasoro 10386* (BCN)	KC212232	–	Riina et al. 2013
*E. epithymoides*	*Efimov & Heida 13* (LE)	GU953742	–	Kryukov et al. 2010
*E. eriophora*	*M. Iranshahr 46720* (IRAN)	KC212238	–	Riina et al. 2013
*E. eugeniae*	*D. Geltman 57a* (LE)	GU979428	–	Kryukov et al. 2010
*E. fauriei*	*Kim & Tho 2001–0002* (–)	EU659767	–	Barres et al. 2011
*E. fanjingshanensis* 1	*G.X. Hu in N. Wei 1* (HIB)	PX646526	PX778798	This study
*E. fanjingshanensis* 2	*C.J. Zhou Q20250528* (HIB)	PX646527	PX778799	This study
*E. fistulosa*	*P. Davis 28230* (LE)	KC212249	–	Riina et al. 2013
*E. flavicoma*	*J. Molero et al. BCN53617* (*duplicate*) (MICH)	JN250152	JN249142	Horn et al. 2012
*E. fragifera Jan*	*Bacic 11126* (IB)	JN010048	–	Frajman and Schönswetter 2011
*E. glabri-flora*	*B. Frajman & P. Schönswetter 12391* (IB)	JN010049	–	Frajman and Schönswetter 2011
*E. gregersenii*	*B. Frajman & P. Schönswetter 12554* (IB)	JN010051	–	Frajman and Schönswetter 2011
*E. haussknechtii*	*H. Haussknecht s.n*. (LE)	KC212269	–	Riina et al. 2013
*E. helioscopia*	*W. Till 4529* (WU)	JN010052	KC212533	Frajman and Schönswetter 2011
*E. hirsuta*	*R. Riina 1769* (*duplicate*) (MICH)	JN250171	JN249161	Horn et al. 2012
*E. hyberna*	*B. Frajman & P. Schönswetter 11436* (IB)	JN010056	–	Frajman and Schönswetter 2011
*E. hylonoma* 1	*Q451* (–)	MH711379	–	NCBI
*E. hylonoma* 2	*TianXH317* (–)	MH808687	–	NCBI
*E. illirica*	*Barres 39* (BC)	HQ900616	–	Barres et al. 2011
*E. jolkinii*	N. Yang 0935 (KUN)	KC212282	KC212548	Riina et al. 2013
*E. lamprocarpa*	*D. Geltman 199* (LE)	KC212294	KC212554	Riina et al. 2013
*E. lathyris*	*J. Walter 4.7.1995* (WU)	JN010059	KC212555	Frajman and Schönswetter 2011
*E. lucorum*	*B.U. Oh s.n*. (–)	EU659771	–	Barres et al. 2011
*E. macrocarpa*	*K. Maroufi 8208* (–)	KC212297	–	Riina et al. 2013
*E. mazandaranica*	*A. Pahlevani & M. Eskandari 55150* (IRAN)	KC212304	–	Riina et al. 2013
*E. mellifera*	*J. Molero 12/2007* (BCN)	KC212306	KC212570	Riina et al. 2013
*E. micractina* 1	*W. Jin 008* (MICH)	KC212308	KC212571	Riina et al. 2013
*E. micractina* 2	*W. Jin 008b* (MICH)	KC212309	–	Riina et al. 2013
*E. montenegrina*	*M. Niketic & al. 11898* (IB)	JN010068	KC212574	Frajman and Schönswetter 2011
*E. nereidum*	*R. Riina 1778* (MICH)	KC212317	KC212579	Riina et al. 2013
*E. oblongata*	*R. Riina 1839* (MICH)	KC212325	KC212587	Riina et al. 2013
*E. orientalis*	*BRUS 1981–12301* (K)	EU659764	–	Barres et al. 2011
*E. pachyrrhiza*	*S. Smirnov et al. s.n*. (LE)	KC212328	–	Riina et al. 2013
*E. palustris*	*B. Frajman & P. Schönswetter 11099* (IB)	JN010073	KC212591	Frajman and Schönswetter 2011
*E. paniculata*	*J. Molero & al. 16/2008* (*duplicate*) (BCN)	KC212330	KC212594	Riina et al. 2013
*E. pekinensis*	*no collector* (–)	AY594263	–	NCBI
*E. phymatosperma*	*S. Zarre et al. 41004* (TUH)	KC212336	KC212599	Riina et al. 2013
*E. pilosa*	D. Geltman 2 (LE)	KC212337	–	Riina et al. 2013
*E. platyphyllos*	*J. Molero s.n*. (BCN)	KC212341	KC212603	Riina et al. 2013
*E. polychroma*	*B. Frajman & P. Schönswetter 11362* (IB)	JN010083	KC212605	Frajman and Schönswetter 2011
*E. polygalifolia*	*J. Molero & A. Rovira BCN53606* (MICH)	KC212344	–	Riina et al. 2013
*E. procera*	*D. Geltman 12* (LE)	GU979433	–	Kryukov et al. 2010
*E. pterococca*	*J. Molero & A. Rovira 20/2007* (*duplicate*) (MICH)	KC212348	KC212611	Riina et al. 2013
*E. purpurea*	*J. Morawetz 315* (MICH)	KC212349	KC212612	Riina et al. 2013
*E. pyrenaica*	*J. Molero & A. Rovira BCN53619* (BCN)	KC212356	KC212614	Riina et al. 2013
*E. rhabdotosperma*	*Y. Roskov 793* (LE)	KC212360	KC212618	Riina et al. 2013
*E. semivillosa*	*M. Wernisch 12476* (WHB)	JN010098	KC212632	Frajman and Schönswetter 2011
*E. sojakii*	*B. Frajman & P. Schönswetter 12416* (IB)	JN010100	–	Frajman and Schönswetter 2011
*E. spathulata*	*G. Rink 5861* (*duplicate*) (NY)	JN250242	JN249229	Horn et al. 2012
*E. spinosa*	*no collector* (–)	EU650602	–	Zecca et al. 2011
*E. squamigera*	*R. Riina 1760* (MA)	KC212387	KC212642	Riina et al. 2013
*E. squamosa*	*D. Geltman 33* (LE)	GU937804	–	Kryukov et al. 2010
*E. stricta*	*B. Frajman & P. Schönswetter 12398* (IB)	JN010104	KC212649	Frajman and Schönswetter 2011
*E. stygiana*	*J. Molero & al. BCN 86911* (BCN)	KC212397	–	Riina et al. 2013
*E. subamplexicaulis*	*S. Smirnov et al. s.n*. (LE)	KC212398	–	Riina et al. 2013
*E. sultan-hasei*	*R. Riina 1568* (MICH)	KC212401	KC212653	Riina et al. 2013
*E. talastavica*	*D. Geltman 206* (LE)	KC212405	–	Riina et al. 2013
*E. texana*	*B. Tharp s.n*. (MICH)	KC212409	–	Riina et al. 2013
*E. transoxana*	*R. Kamelin 1717* (LE)	GU979425	–	Kryukov et al. 2010
*E. uliginosa*	*J. Molero & A. Rovira BCN53605* (BCN)	KC212418	KC212669	Riina et al. 2013
*E. valdevillosocarpa*	*I. Zhilkina s.n*. (LE)	GU979431	–	Kryukov et al. 2010
*E. valerianifolia*	*W. Gutermann 34627* (Herb. Gutermann)	JN010109	–	Frajman and Schönswetter 2011
*E. velenovskyi*	*B. Frajman & P. Schönswetter 11345* (IB)	JN010113	KC212674	Frajman and Schönswetter 2011
*E. verrucosa*	*B. Frajman & P. Schönswetter 11771* (IB)	JN010115	–	Frajman and Schönswetter 2011
*E. wallichii*	*W. Jin 009* (MICH)	KC212426	KC212683	Riina et al. 2013
*E. xiangxiui* 1	*Q. Yu LX20051201* (HIB!)	MZ468551	MZ494740	Yu et al. 2022
*E. xiangxiui* 2	*Q. Yu LX20051202* (HIB!)	MZ468552	–	Yu et al. 2022

### Molecular phylogenetic analyses

All ITS and *ndhF* sequences were aligned using MAFFT v7 (Katoh and Standley 2013) under default parameters, and poorly aligned regions were removed with TrimAl (Capella-Gutiérrez et al. 2009). These steps were performed within the PhyloSuite v1.2.2 platform (Zhang et al. 2020). The trimmed alignments were visually inspected and manually adjusted, when necessary, in Geneious Prime v2025.0.3 (Kearse et al. 2012). Phylogenetic analyses were conducted using both Maximum Likelihood (ML) and Bayesian Inference (BI) frameworks, implemented in IQ-TREE (Nguyen et al. 2015) and MrBayes (Ronquist et al. 2012), respectively. The best-fitting partitioning scheme and substitution models were selected with PartitionFinder 2 (Lanfear et al. 2017), based on the Akaike Information Criterion (AIC) for ML and the Bayesian Information Criterion (BIC) for BI analyses. Methods for phylogenetic reconstruction followed Wei et al. (2021) and Yu et al. (2022). The resulting trees were visualized and annotated using iTOL (https://itol.embl.de/) (Letunic and Bork 2021).

## Results

### Morphological study

*Euphorbia fanjingshanensis* is morphologically closest to *E. micractina* Boiss. (1862: 127) and *E. pekinensis* Rupr. (1859: 239), but is distinguishable by a combination of morphological characters. *Euphorbia fanjingshanensis* possesses vertical, cylindrical roots ca. 10 cm long and 3–5 mm in diam. Whereas *E. micractina* has tuberous or rhizomatous roots, and *E. pekinensis* has longer and thicker roots, 20–30 cm long and 6–14 mm thick, which are vertical, cylindrical, and sometimes branched. *Euphorbia fanjingshanensis* is an erect herb up to 75 cm tall, with up to eight pubescent to subglabrous stems. In contrast, *E. micractina* has glabrous stems that are unbranched apically, whereas *E. pekinensis* has stems 40–90 cm tall that are solitary or much branched from the base and range from pubescent to glabrous. The leaves of *E. fanjingshanensis* are 4–10 × 1–2 cm, lanceolate, green, with entire margins and acute apices; whereas those of *E. micractina* are distinctly smaller, 0.7–2.0 × 0.5–1 cm, oblong to elliptic, with rounded apices; and those of *E. pekinensis* are usually 3–7(–9.5) × 0.7–1.7(–2.4) cm, usually elliptic, and acuminate or acute apices. In *E. fanjingshanensis*, the peduncle, cyathophylls, involucre, and style are usually reddish to crimson, whereas these structures are less reddish in *E. micractina* and *E. pekinensis*. Besides, *E. fanjingshanensis* has moderately warted capsules, in contrast to the relatively sparse, spiny-tuberculate capsules of *E. micractina*, whereas those of *E. pekinensis* are only sparsely tuberculate. Additional morphological differences among these three species are presented in Table 2.

**Table 2.** Morphological comparison among *E. fanjingshanensis*, *E. micractina*, and *E. pekinensis*.

**Character**	***E. fanjingshanensis***	***E. micractina***	***E. pekinensis***
**Root size**	Ca. 10 cm long, 3–5 mm thick	10–12 cm long; tubers 6–12 mm thick; rhizomes 2–3 mm thick	20–30 cm long, 6–14 mm thick
**Root type**	Vertical, cylindric	Tuberous or rhizomatous	Vertical, cylindric, sometimes branched
**Stem**	Clustered (≤8), upper part usually unbranched	Clustered (≤4), upper part usually unbranched	Solitary or clustered, upper parts 4–5-branches
**Leaf size**	4–10 × 1–2 cm	0.7–2.0 × 0.5–1 cm	3–7(–9.5) × 0.7–1.7(–2.4) cm
**Leaf shape**	Lanceolate	Oblong to elliptic	Usually, elliptic
**Leaf apex**	Acute	Rounded	Acuminate or acute
**Involucre**	Abaxially reddish, pale green to red	Abaxially reddish, pale green to red	Abaxially pale green
**Cyathophylls**	Green or with reddish margins	Green to red	Yellow or green
**Gland number**	4, sometimes 5	4	4
**Gland shape**	Reniform	Rounded to transversely elliptic	Reniform
**Style color**	Red to crimson, sometimes green with flushed red apices	Green to yellow, sometimes red	Green to yellow, sometimes red
**Capsule**	Moderately tuberculate, with short tubercles	Sparsely softly spiny or tuberculate along angles	Sparsely tuberculate
**Seed size**	Ca. 2.5 × 2 mm	Ca. 2 × 1.5 mm	Ca. 2.5 × 1.5–2 mm
**Seed shape**	Ovoid-globose	Broadly ovoid	Long-globose
**Seed color**	Dark brown	Gray-brown	Dark brown or slightly shiny

### Phylogenetic analysis

**Figure 1.**
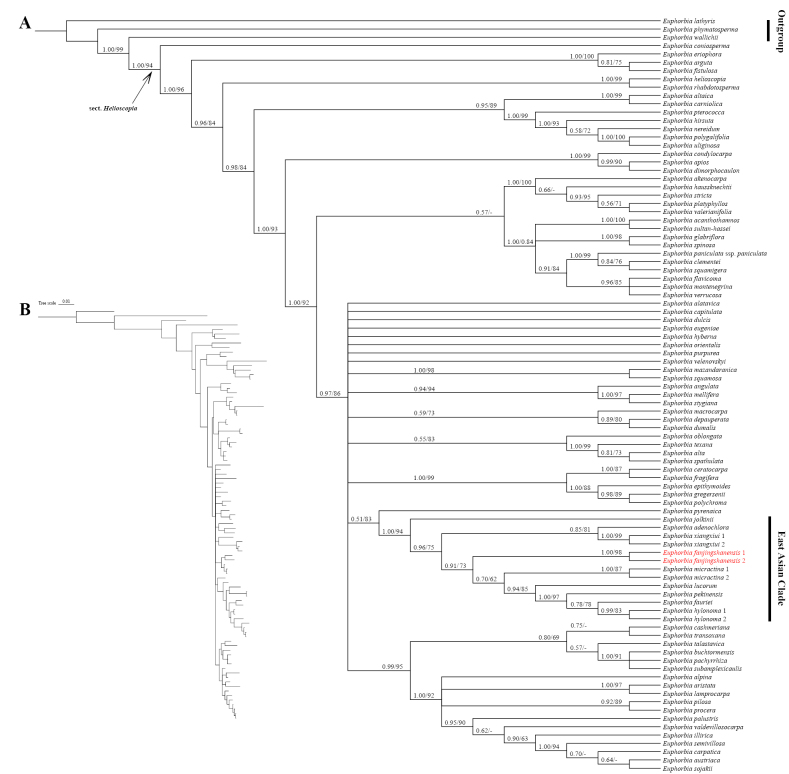
**A**. Bayesian inference (BI) tree of *Euphorbia* based on combined nuclear **ITS** and plastid ***ndhF*** sequences. Bootstrap support (BS) and Bayesian posterior probability (PP) values are indicated on the nodes as **BS/PP**. The new species *E. fanjingshanensis* is highlighted in **bold red**. **B**. The same tree as **A**, but showing branch lengths proportional to nucleotide substitutions per site.

The ILD test indicated no significant incongruence between the ITS and *ndhF* datasets (p = 0.26, > 0.01), supporting their combination for subsequent phylogenetic inference. The best-fit substitution models selected for the ITS and *ndhF* partitions were SYM + I + G and TVM + I + G, respectively, and these models were used in both the maximum likelihood (ML) and Bayesian inference (BI) analyses. The ML and BI analyses produced largely identical topologies, with only a few minor incongruences (Fig. 1). *Euphorbia* sect. *Helioscopia* was recovered as monophyletic with strong support (BS = 94%; PP = 1.0). The two accessions of *E. fanjingshanensis* clustered together with full support (BS = 98%; PP = 1.0) and were deeply nested within *E.* sect. *Helioscopia*. *Euphorbia fanjingshanensis* was recovered as sister to a clade comprising five East Asian species (*E. micractina*, *E. lucorum*, *E. pekinensis*, *E. fauriei*, and *E. hylonoma*), with moderate support (BS = 73%; PP = 0.91).

## Discussion

Morphological and molecular evidence clearly support the status of *Euphorbia fanjingshanensis* as a distinct species. Although this species is morphologically similar to *E. micractina* and *E. pekinensis*, it can be effectively distinguished from these two species by a unique combination of morphological features. The key characteristics of *E. fanjingshanensis* include its lanceolate leaves with acute apices, distinctive synflorescence characters, and ovoid-globose dark brown seeds. In particular, the capsules of *E. fanjingshanensis* are moderately tuberculate, whereas those of *E. micractina* and *E. pekinensis* exhibit sparser tuberculation. In addition, the seeds of *E. fanjingshanensis* are ovoid-globose and dark brown, whereas those of *E. micractina* are broadly ovoid and gray-brown, and those of *E. pekinensis* are elongate-globose, dark brown to slightly shiny, with a pale ventral stripe. These differences serve as the primary distinguishing features of *E. fanjingshanensis* from other species. Molecular phylogenetic analyses based on combined ITS and *ndhF* sequences support the placement of *E. fanjingshanensis* within *Euphorbia* sect. *Helioscopia* of subg. *Esula*. The species belongs to the East Asian clade, which was defined by Geltman (2015) and Yu et al. (2022).

*Euphorbia fanjingshanensis* is primarily distributed in the transitional zone between the Yunnan-Guizhou Plateau and the Wuling Mountains, suggesting strong adaptability. The species was first recorded in the Fanjingshan area of Guizhou Province, and subsequent studies have also identified it in the Chuankongdong Cave in Changyang Tujia Autonomous County, Yichang City, Hubei Province. Although these localities are situated in different administrative regions, both are within the transitional zone between the Yunnan-Guizhou Plateau and the Wuling Mountains. This pattern suggests that the species may possess a wider distribution than currently documented. The integration of morphological observations and molecular phylogenetic analyses has improved our understanding of the taxonomic identity and evolutionary placement of *E. fanjingshanensis*. These findings also provide additional insights into the diversity and biogeographic patterns of the East Asian clade within *Euphorbia*. Future research is expected to further uncover more evolutionary relationships between this species and its closely related taxa.

### Taxonomic treatment

#### Euphorbia fanjingshanensis

Taxon classificationPlantaeMalpighialesEuphorbiaceae

N.Wei, Lei Shi & Q.F.Wang sp. nov.

B82CD742-7574-5D84-B164-AC6E58981DC2

urn:lsid:ipni.org:names:77383765-1

##### Type.

China • Guizhou: Tongren City, Fanjingshan Mountain, near the summit, 27.9143, 108.6922, 2263 m, 25 July 2024, *N.Wei & Z.H.Xin WN16* (holotype: HIB!; isotypes: HIB!, PE!)

##### Diagnosis.

*Euphorbia fanjingshanensis* is most similar to *E. micractina* and *E. hylonoma*, but differs from *E. micractina* and *E. hylonoma* mainly by its lanceolate leaves with pointed tips, crimson to reddish peduncles and raylet-leaves, moderately tuberculate capsules, and dark brown ovoid-globose seeds (Table 2).

##### Description.

Herbs, erect, up to 75 cm tall. ***Root*** thick, up to more than 10 cm × 3–5 mm. ***Stems*** 3–7 mm thick, in clusters of up to 8, upper parts usually unbranched, pilose or glabrous. ***Leaves*** lanceolate, 4–10 × 1–2 cm, both surfaces glabrous; adaxial surface green, abaxial sometimes light purple; base attenuate, apex acute, margin entire; midrib prominent abaxially, often grooved adaxially; lateral veins 6–10 pairs, inconspicuous. ***Synflorescences*** arranged in a terminal pseudumbel, usually also with long-pedunculate dichasial cymes from axils; cymes 2–4-forked; primary involucral leaves 5, similar to normal leaves, margin entire, base rounded, apex rounded, primary rays 2.0–5.5 cm long; ***raylet-leaves and subcyathial raylet-leaves*** 2 or 3, triangular-ovate, 0.7–1.7 × 0.5–1.5 cm, green with reddish margin to red, sessile. ***Cyathia*** sessile; ***involucre*** campanulate, ca. 2.5 × 2.5–3.5 mm, lobes pink to red, triangular to linear-triangular, glabrous; glands 4, occasionally 5, pale yellow to dark brown, reniform. ***Male flowers*** numerous, exserted from the involucre, 1.0–1.5 mm. ***Pollen grains*** are radially symmetrical and 3-colporate, ca. 30 × 25 μm, nearly oblate in equatorial view with a longer equatorial axis and a shorter polar axis, trilobed-circular in polar view, and ornamented with separate perforations ranging from circular and broadly elliptic to linear-oblong; ***Female flower*** with ovary globose, tuberculate; ***styles*** connate for less than half their length, red to crimson, sometimes green with flushed red apex, persistent but easily fallen; ***style arms*** bifid. ***Capsule*** globose, ca. 4.5 × 4.0–4.5 mm, moderately tuberculate with short tubercles ca. 1 mm long. ***Seeds*** ovoid-globose, ca. 2.5 × 2 mm, dark brown, slightly shiny, smooth, adaxially striate; ***caruncle*** peltate, shortly stipitate.

**Figure 2.**
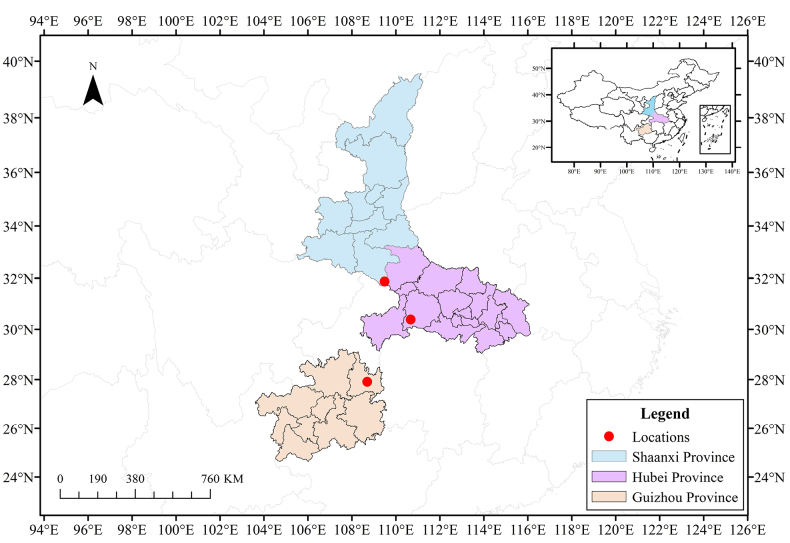
Distribution map of *Euphorbia fanjingshanensis* in China. The red dots indicate the collection localities of the species: one in Fenggao County, Ankang City, Shaanxi Province, one in Fanjingshan National Nature Reserve, Tongren City, Guizhou Province, and the other in Changyang County, Hubei Province. Inset map shows the positions of Shaanxi, Guizhou and Hubei provinces within China.

**Figure 3.**
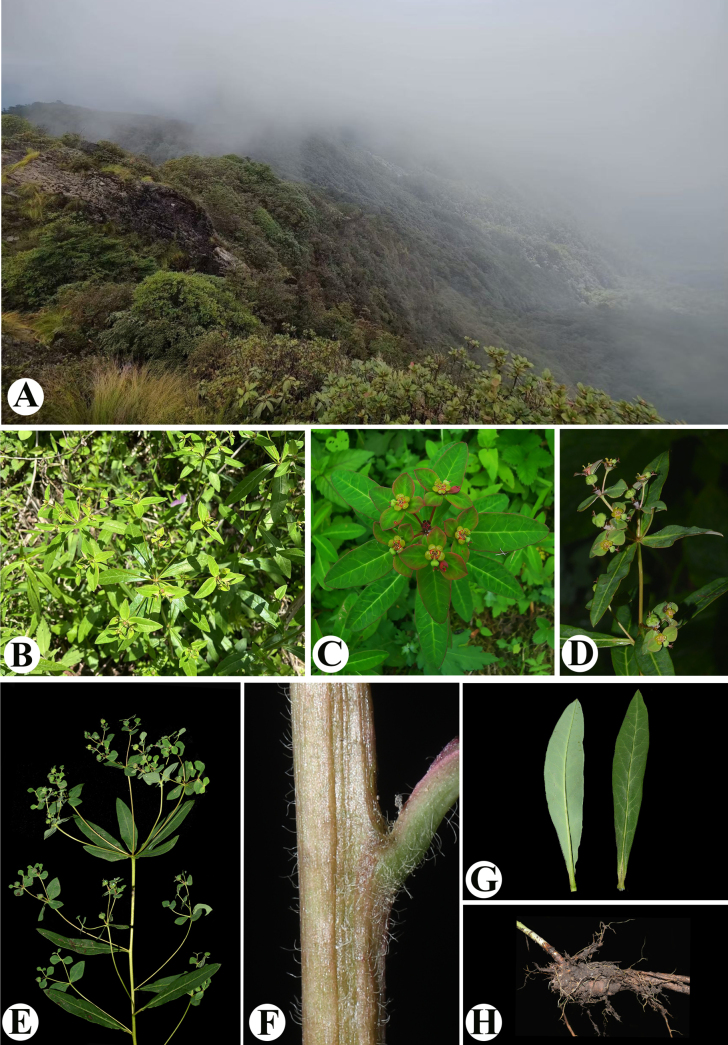
*Euphorbia fanjingshanensis*. **A**. Habitat on the summit of Mount Fanjing, Guizhou Province, within the cloud-forest zone; **B**. Flowering branch; **C**. Terminal umbel-like synflorescence with adaxial and abaxial leaf surfaces; **D**. Habit of a flowering individual; **E**. Whole plant showing branching pattern and leaf arrangement; **F**. Stem indumentum; **G**. Adaxial and abaxial leaf surfaces; **H**. Tuberous root. Scale bars: 2 cm (**E, G, H**); 2 mm (**F**).

**Figure 4.**
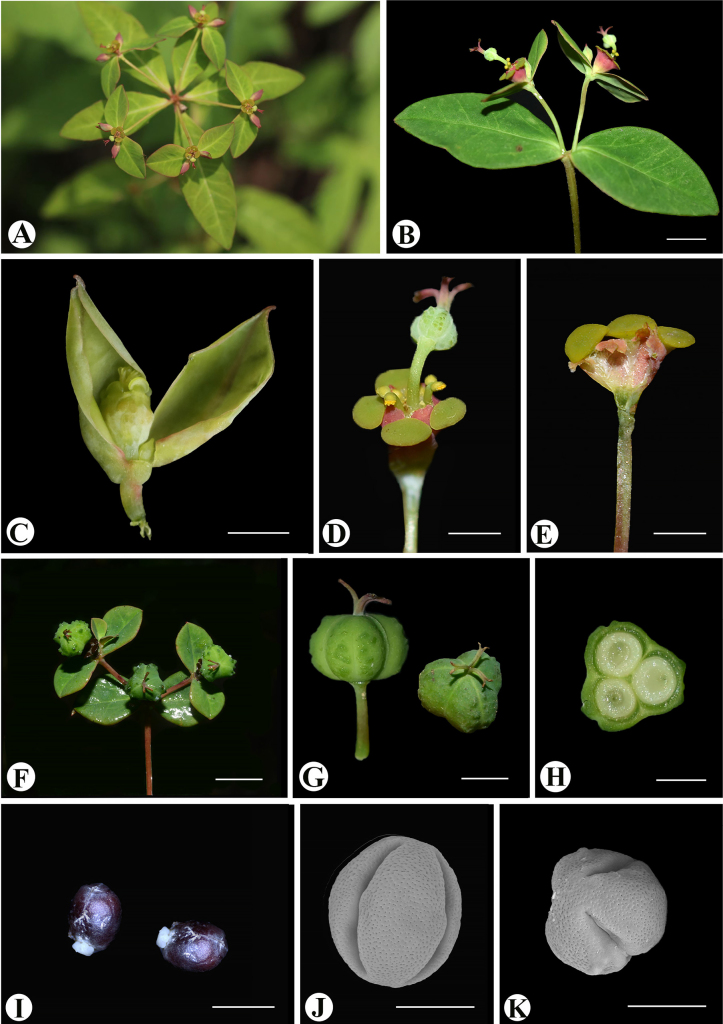
*Euphorbia fanjingshanensis*. **A**. Cyathia on terminal branches; **B**. Cyathia on slender peduncle; **C**. Longitudinally dissected cyathium showing involucre, glands, lobes, and enclosed male flowers; **D**. Male flower with exserted stamens; **E**. Glands and lobes of the involucre; **F**. infructescence with developing capsules; **G**. Capsules showing external ornamentation and internal locules; **H**. Transverse section of capsule showing three locules; **I**. Seeds; **J**. Pollen grain under SEM, equatorial view; **K**. Pollen grain under SEM, polar view. Scale bars: 5 mm (**B, F**); 1 mm (**C**); 2 mm (**D, E, G, H, I**); 15 µm (**J, K**).

##### Distribution and ecology.

The species grows in humid and open rocky slopes. It is currently known from the three localities in southwestern and central China.

##### Phenology.

Flowering observed from May to July.

##### Etymology.

The species epithet, ‘*fanjingshanensis*’, originates from one of its type localities, Fanjingshan Mountain.

##### Paratypes.

• China. Guizhou Province, Tongren City, Fanjingshan Mountain, near the summit; ca. 2200 m; 24 July 1941; s. *coll. 967* (PE!). • China. Guizhou Province, Tongren City, Fanjingshan Mountain, near the summit; ca. 1650 m; 4 May 1959; *Qianbei Expedition* 654 (NAS!). • China. Guizhou Province, Tongren City, Fanjingshan Mountain, near the summit; ca. 1650 m; 4 May 1959; *T.P. Zhu & Z.F. Liu 654* (KUN!). • China. Guizhou Province, Tongren City, Fanjingshan Mountain, near the summit; 2269.9 m; 22 July 2016; *D.W. Lu 522222160722022LY* (GZTM!). • China. Guizhou Province, Tongren City, Fanjingshan Mountain, near the summit; ca. 2200 m; 9 June 2023; *G.X. Hu in N. Wei 1* (HIB!). • China. Hubei Province, Yichang City, Changyang Tujia Autonomous County, Chuankongdong Cave, 30.3858°N, 110.6761°E; ca. 400 m; 28 May 2025; *C.J. Zhou Q20250528* (HIB!). • China. Shaanxi Province, Langao County; ca. 2300 m; 19 July 2008; *Bashan Collecting Team 1838* (PE!).

##### Conservation status.

*Euphorbia fanjingshanensis* is given a preliminary assessment of Least Concern (LC). The species is currently known from three localities in China: Fanjingshan in Guizhou, Chuankongdong Cave in Changyang, Hubei, and Langao in Shaanxi. A quick evaluation using GeoCAT indicates an Area of Occupancy (AOO) of ca. 12 km^2^ and an Extent of Occurrence (EOO) of approximately 31,385 km^2^. Although the distribution is relatively restricted, all known populations occur within protected areas or intact montane habitats, with stable population sizes and no observed major threats. Therefore, the species is currently assessed as LC under the IUCN Red List criteria. (IUCN 2001).

## Supplementary Material

XML Treatment for Euphorbia fanjingshanensis

## Additional information

### Artificial Intelligence (AI) use

The authors accept full responsibility for the content of the manuscript, including the disclosure of any use of AI.

No AI tools were used in the preparation of this manuscript.

### Author ORCIDs

R.T. Ratsiferanarivo https://orcid.org/0009-0003-1006-100X

X.-D. Qiu https://orcid.org/0009-0007-2464-6921

N. Wei https://orcid.org/0000-0002-0313-3912

Q.-F. Wang https://orcid.org/0000-0001-9143-8849

### Data availability

All of the data that support the findings of this study are available in the main text.
